# Comparative real-world safety profiles of selective β1-adrenergic receptor blockers: A multi-database pharmacovigilance disproportionality study

**DOI:** 10.1097/MD.0000000000050763

**Published:** 2026-09-25

**Authors:** Weijie Ren, Haosen Zhao, Zhenzhong Wang, Jixin Li, Zelong Niu, Ruiling Zhou, Zhijie Cui, Zezhou Zhang, Peili Wang

**Affiliations:** a Department of Cardiology, Affiliated Hospital of Shanxi University of Chinese Medicine, Shanxi University of Chinese Medicine, Taiyuan, Shanxi, China; b Department of Integrative Cardiology, Xiyuan Hospital, China Academy of Chinese Medical Sciences, Beijing, China; c Department of Rehabilitation Medicine, PKUCare CNOOC Hospital, Tianjin, China.

**Keywords:** adverse drug events, pharmacovigilance, real-world evidence, safe medication recommendations, selective β1-blockers, sex differences

## Abstract

Selective β1-blockers are fundamental in managing hypertension, coronary artery disease, and heart failure. Yet, despite their widespread use, practical guidance on safe and individualized prescribing remains limited. Existing evidence is largely derived from controlled clinical trials, which may not fully capture drug-specific adverse events, sex-related susceptibility, or early-onset risks observed in routine clinical practice. To generate real-world, evidence-based recommendations for the safe use of selective β1-blockers, we compared the adverse event profiles of metoprolol, bisoprolol, and atenolol using 2 large pharmacovigilance systems. Adverse drug events reported for the 3 agents between 2004 and Q2 2025 were retrieved from the US Food and Drug Administration Adverse Event Reporting System, a spontaneous-reporting system, and compared with reports from the Canadian Vigilance Adverse Reaction Online Database. Disproportionality analyses, preferred term mapping, exploratory sex-stratified comparisons, and time-to-onset modeling were conducted to characterize shared reporting patterns, drug-specific signals, and potential patient-level modifiers. Across all 3 agents, disproportionate reporting was observed for a common cardiovascular spectrum that included bradycardia, conduction abnormalities, heart failure, and blood pressure instability. Metoprolol showed prominent reporting signals for BRASH syndrome and neuropsychiatric and suicide-related events. Bisoprolol showed signals for bradyarrhythmia, hyperkalemia, and acute kidney injury. Atenolol showed reporting patterns involving blood pressure perturbations, electrolyte imbalance, and interaction-related events. Exploratory sex-stratified analyses identified differences in the reporting distributions of several PT = preferred terms for metoprolol and atenolol, whereas bisoprolol showed a more balanced distribution. All 3 agents displayed early-failure time-to-onset patterns, supporting closer monitoring after treatment initiation. This real-world pharmacovigilance assessment identifies drug-specific patterns of disproportionate reporting that may inform hypothesis generation and individualized monitoring. Because spontaneous-reporting data cannot establish incidence or causality, the findings should complement, rather than replace, clinical judgment and confirmation in controlled or longitudinal data sources.

## 1. Introduction

Cardiovascular disease remains the leading cause of mortality and disability worldwide, and the long-term management of chronic conditions such as hypertension, coronary artery disease, and heart failure places increasingly stringent demands on the safety and efficacy of pharmacotherapy.^[1]^ Selective β1-adrenergic receptor blockers, by specifically antagonizing cardiac β1 receptors and thereby reducing heart rate and myocardial oxygen consumption, are widely used in the treatment of hypertension, arrhythmias, heart failure, and coronary artery disease, and constitute a cornerstone of cardiovascular drug therapy.^[2]^ Among them, metoprolol, bisoprolol, and atenolol are the most commonly prescribed agents. Their efficacy in cardiovascular disease has been well established by robust evidence, and they have been incorporated into multiple international guidelines as key components of long-term maintenance therapy.^[3,4]^

Although selective β1-blockers exhibit substantially greater cardiac selectivity than nonselective β-blockers, with relatively fewer adverse effects such as bronchospasm and peripheral vasoconstriction, clinical practice and real-world reports indicate that they can still induce multisystem adverse drug events (ADEs).^[5]^ Typical ADEs include bradycardia, hypotension, dizziness, fatigue, impaired consciousness, and electrolyte disturbances. Some events may lead to hospitalization, dose adjustment, or even drug discontinuation, thereby compromising long-term adherence and clinical outcomes.^[6]^ Importantly, there are marked differences among β1-blockers in pharmacokinetics, lipophilicity, half-life, and routes of elimination – for example, metoprolol is highly lipophilic with strong central nervous system penetration;^[7]^ bisoprolol has higher β1 selectivity and a more prolonged duration of action;^[8]^ and atenolol has low lipophilicity and is predominantly eliminated via the kidneys. These distinctions may result in heterogeneous safety profiles in real-world settings, yet systematic comparative studies remain scarce.^[9]^

In addition to drug-specific characteristics, patient-level factors profoundly influence the risk of ADEs associated with β1-blockers. For instance, older adults, due to diminished autonomic regulation, are more prone to bradycardia and falls; patients with impaired renal function treated with atenolol may be at increased risk of ADEs due to drug accumulation; and variations in body weight, sex, polypharmacy, and underlying chronic diseases may further modify ADE occurrence.^[10,11]^ However, existing studies often focus on single risk factors or individual agents, and there is a lack of large-scale real-world comparative analyses encompassing the 3 major β1-blockers.

Real-world data (RWD) provide an important complement to traditional clinical trials in drug safety research. The US Food and Drug Administration Adverse Event Reporting System (FAERS), one of the largest pharmacovigilance databases globally, captures post-marketing safety information across countries, patient populations, and complex clinical contexts, thereby reflecting the actual safety performance of marketed drugs.^[12]^ Meanwhile, regional databases such as the Canadian Vigilance Adverse Reaction Online Database (CVARDD) can serve as external validation sources, enhancing the robustness and reliability of potential ADE signals. Integrated analyses across multiple databases enable signal corroboration, cross-population risk comparison, and in-depth exploration of temporal patterns of adverse reactions.^[13]^

Against this background, the present study uses FAERS and CVARDD to compare the reporting spectra and signal strengths of ADEs associated with metoprolol, bisoprolol, and atenolol. These agents were selected because they are widely used selective β1-blockers and differ meaningfully in lipophilicity, β1-selectivity, duration of action, metabolism, and renal elimination, enabling a clinically relevant comparison of pharmacologically distinct agents within the same therapeutic class. We further examine time-to-onset (TTO) characteristics and exploratory sex-stratified reporting patterns. The aim is to characterize potential safety signals and generate hypotheses that may support individualized monitoring and future confirmatory research.

## 2. Materials and methods

### 2.1. Data sources

The ADE data used in this study were obtained from the FAERS. FAERS has been publicly accessible since 2004 and collects spontaneous reports of adverse events from diverse sources, including healthcare professionals, pharmaceutical manufacturers, and patients. To investigate ADEs associated with the selective β1-adrenergic receptor blockers metoprolol, bisoprolol, and atenolol, all available records from database inception through the second quarter (Q2) of 2025 were extracted. The data were then imported into MySQL version 15.0 (Oracle Corporation) and processed using Navicat Premium 15 to support comprehensive analysis.

### 2.2. Data extraction and processing

Drug names for metoprolol, bisoprolol, and atenolol were standardized using Medex_UIMA_1.8.3. The FAERS data were preprocessed using SAS and MySQL. Duplicate reports in the DEMO table were identified using the FAERS case identifier, and only the most recent version of each case was retained. Metoprolol, bisoprolol, and atenolol were defined as suspected drugs. Adverse events were mapped to preferred terms (PTs) and system organ classes (SOCs) using the Medical Dictionary for Regulatory Activities, version 27.1 (International Council for Harmonisation [ICH]; maintained and distributed by MedDRA MSSO). Patient characteristics, including age, sex, body weight, reporting country, indication, and reporting year, were extracted when available. Concomitant medications were retained as descriptive report-level information but were not included in patient-level adjusted analyses because of incomplete and inconsistent reporting. Missing values were not imputed and were summarized as missing in the descriptive analysis. The complete workflow for data extraction, processing, and analysis is presented in Figure 1.

**Figure 1.**
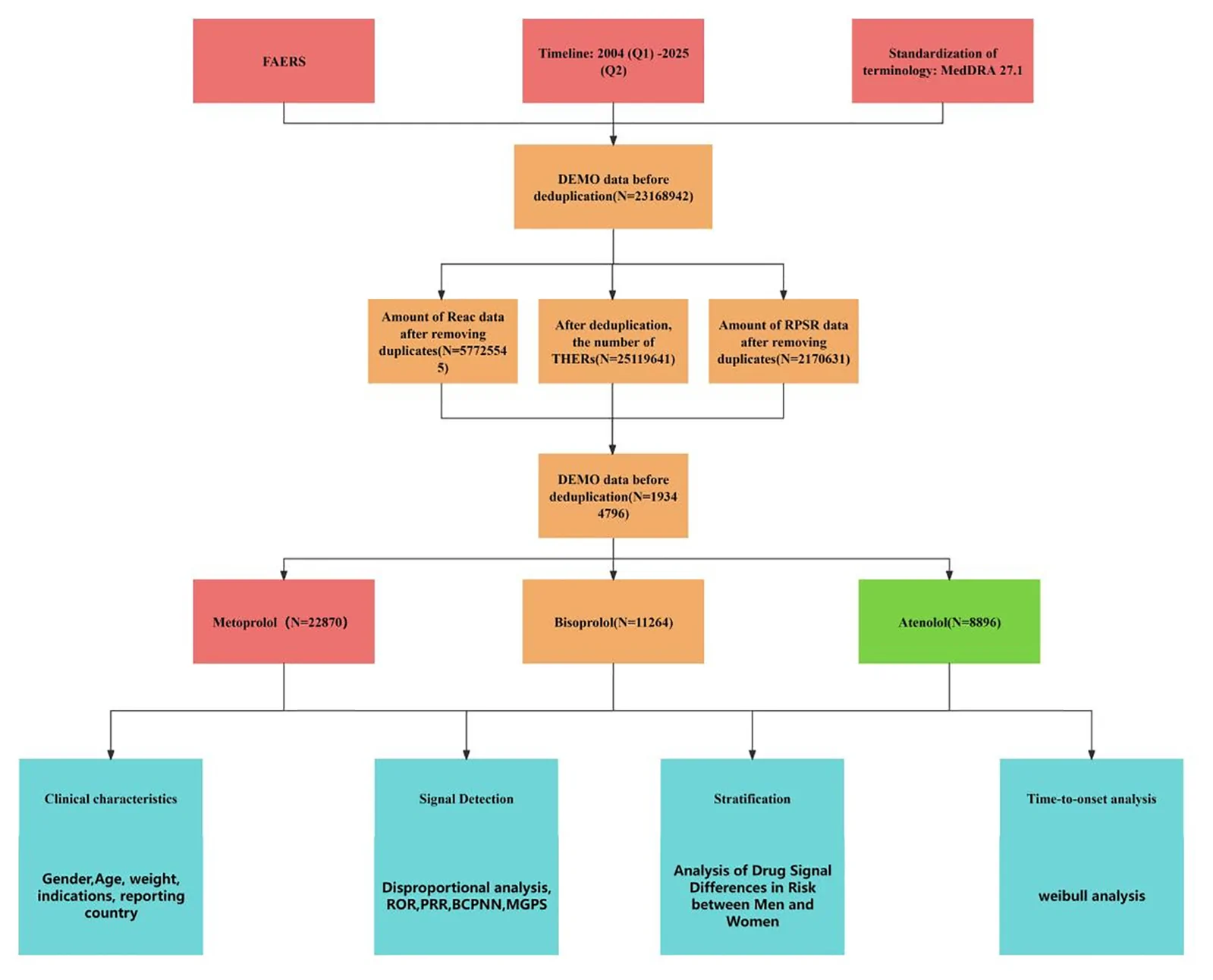
Analytical workflow for adverse event assessment of the selective β1-blockers metoprolol, bisoprolol, and atenolol.

### 2.3. Data-mining algorithms

To identify potential associations between the selective β1-adrenergic receptor blockers (metoprolol, bisoprolol, and atenolol) and ADEs, disproportionality analyses were conducted. Disproportionality analysis is a core pharmacovigilance method that evaluates the relationship between a drug and an ADE by comparing the frequency ratio of reported events in exposed versus unexposed populations using a 2 × 2 contingency table.

In this study, 4 commonly used disproportionality algorithms were applied to detect safety signals: the reporting odds ratio (ROR), proportional reporting ratio (PRR), Bayesian confidence propagation neural network, and multi-item gamma-Poisson shrinker.^[14-16]^ Using frequentist and Bayesian algorithms with complementary statistical properties was intended to improve signal robustness and reduce dependence on any single method. ROR and proportional reporting ratio provide readily interpretable frequentist measures, whereas Bayesian confidence propagation neural network and MGPS apply shrinkage that can stabilize estimates for sparse reports.

The formulas and threshold criteria for all 4 algorithms are provided in Supplementary Material 1, Supplemental Digital Content 1. Statistical analyses were performed using R software. Values exceeding the prespecified thresholds were interpreted as signals of disproportionate reporting. Their magnitudes do not represent incidence, causal effects, or direct comparisons of clinical danger between drugs.

## 3. Results

### 3.1. Demographic characteristics of patients reporting adverse events related to selective β1-blockers

From the time of drug inclusion in the FAERS database through the second quarter of 2025, a total of 22,870 adverse event reports for metoprolol, 11,264 for bisoprolol, and 8896 for atenolol were identified.

For metoprolol, baseline characteristics showed that the number of female patients substantially exceeded that of male patients (48.7% vs 38.6%). Among reports with available age information, the most common age group was 65 to 85 years (29.8%). Body weight was concentrated in the 50 to 100 kg range (22.9%), although weight data were missing for 70.3% of cases. The most frequently reported indication was hypertension (10.73%). Reports primarily originated from the United States (60.9%), Germany (9.8%), and Canada (2.6%).

For bisoprolol, the number of female patients was slightly higher than that of males (46.7% vs 42.3%). Among cases with documented age, the most common age group was 65 to 85 years (44.2%). Body weight was mainly distributed between 50 and 100 kg (26.1%), but weight data were missing for 67.9% of patients. The predominant indication was hypertension (11.43%). Reports were mainly from France (23.1%), Great Britain (21.5%), and Germany (13.5%).

For atenolol, the proportion of female patients substantially exceeded that of males (53.5% vs 38.9%). Among reports with age information, the most common age group was 65 to 85 years (34.3%). Body weight was mostly within 50 to 100 kg (24.3%), though 69.6% of cases lacked weight data. The leading indication was hypertension (17.23%). Reports primarily originated from the United States (42.1%), France (10.9%), and Italy (10.1%). Detailed demographic information is presented in Table 1.

**Table 1:** Clinical characteristics of metoprolol, bisoprolol, and atenolol adverse event reports from the FAERS database (Storage time - Q2 2025).

Characteristics	Metoprolol		Bisoprolol		Atenolol	
Number of events	22,870		11,264		8896	
Gender, number (%)						
Female	11,130 (48.7%)		5261 (46.7%)		4756 (53.5%)	
Male	8832 (38.6%)		4768 (42.3%)		3464 (38.9%)	
Miss	2908 (12.7%)		1235 (11.0%)		676 (7.6%)	
Age, number (%)						
Median age	65.9		73.0		66.0	
<18	494 (2.2%)		204 (1.8%)		203 (2.3%)	
18–65	6700 (29.3%)		2544 (22.6%)		3001 (33.7%)	
65–85	6810 (29.8%)		4977 (44.2%)		3048 (34.3%)	
>85	1054 (4.6%)		1339 (11.9%)		515 (5.8%)	
Miss	7812 (34.2%)		2200 (19.5%)		2129 (23.9%)	
Weight (KG), number (%)						
<50	584 (2.6%)		295 (2.6%)		197 (2.2%)	
50–100	5228 (22.9%)		2935 (26.1%)		2163 (24.3%)	
≥100	988 (4.3%)		387 (3.4%)		348 (3.9%)	
Miss	16,070 (70.3%)		7647 (67.9%)		6188 (69.6%)	
Top 5 indication, number (%)	Hypertension	(4891, 10.73%)	Hypertension	(2968, 11.43%)	Hypertension	(3290, 17.23%)
	Atrial fibrillation	(1428, 3.13%)	Atrial fibrillation	(1438, 5.54%)	Atrial fibrillation	(389, 2.03%)
	Cardiac disorder	(522, 1.14%)	Cardiac failure	(773, 2.98%)	Diabetes mellitus	(289, 1.51%)
	Arrhythmia	(520, 1.14%	Type 2 diabetes mellitus	(268, 1.03%)	Blood cholesterol increased	(214,1.12%)
	Cardiac failure	(412, 0.90%)	Myocardial ischaemia	(258, 0.99%)	Depression	(204, 1.07%)
Top 5 reported countries, number (%)	United States	(13927, 60.9%)	France	(2601, 23.1%)	United States	(3746, 42.1%)
	Germany	(2241, 9.8%)	Great Britain	(2422, 21.5%)	France	(974, 10.9%)
	Canada	(584, 2.6%)	Germany	(1515, 13.5%)	Italy	(895, 10.1%)
	China	(528, 2.3%)	Italy	(1514, 13.4%)	United Kingdom	(559, 6.3%)
	Netherlands	(506, 2.2%)	Spain	(489, 4.3%)	Brazil	(535, 6.0%)

The report populations differed across drugs in age, indication, country, and missingness. These descriptive differences are relevant potential sources of confounding and reporting heterogeneity; the unadjusted disproportionality analyses should therefore not be interpreted as direct head-to-head estimates of clinical safety.

Figure 2 illustrates clear temporal and inter-drug variations in adverse event reporting patterns among the 3 β1-blockers. For metoprolol, reports increased rapidly after 2008, peaking between 2014 and 2018 (highest in 2018). Although reports declined slightly afterward, they remained at a relatively high level overall. For bisoprolol, reporting volume was low between 2004 and 2013, then rose markedly after 2014, peaking during 2018 to 2020, before gradually decreasing. For atenolol, moderate-to-high report counts were observed during 2008 to 2015, with peaks in 2014 to 2015, followed by a continuous downward trend, reaching low levels by 2023 to 2025. Overall, metoprolol consistently had the highest number of adverse event reports throughout the observation period, followed by bisoprolol, while atenolol showed the lowest and steadily declining reporting frequency.

**Figure 2.**
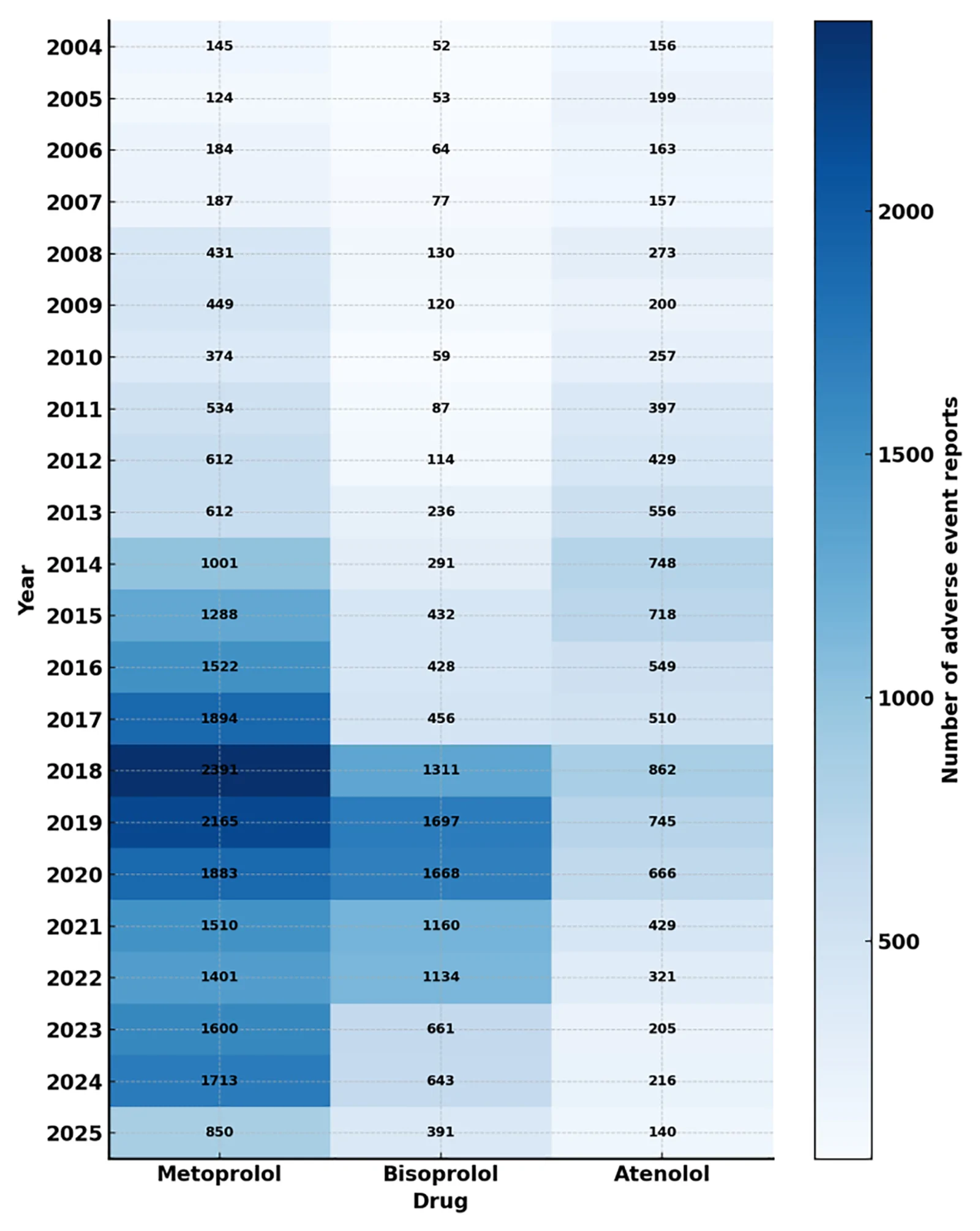
Reporting trends of adverse events associated with selective β1-blockers. SOC = system organ class.

### 3.2. SOC-level signal analysis

Figure 3 presents the SOC-level safety signals for the 3 selective β1-adrenergic receptor blockers. For metoprolol, strong disproportionality signals were observed in cardiac disorders (ROR = 4.79) and vascular disorders (ROR = 2.53). Moderate elevations were also noted in pregnancy, puerperium and perinatal conditions and congenital, familial and genetic disorders.

**Figure 3.**
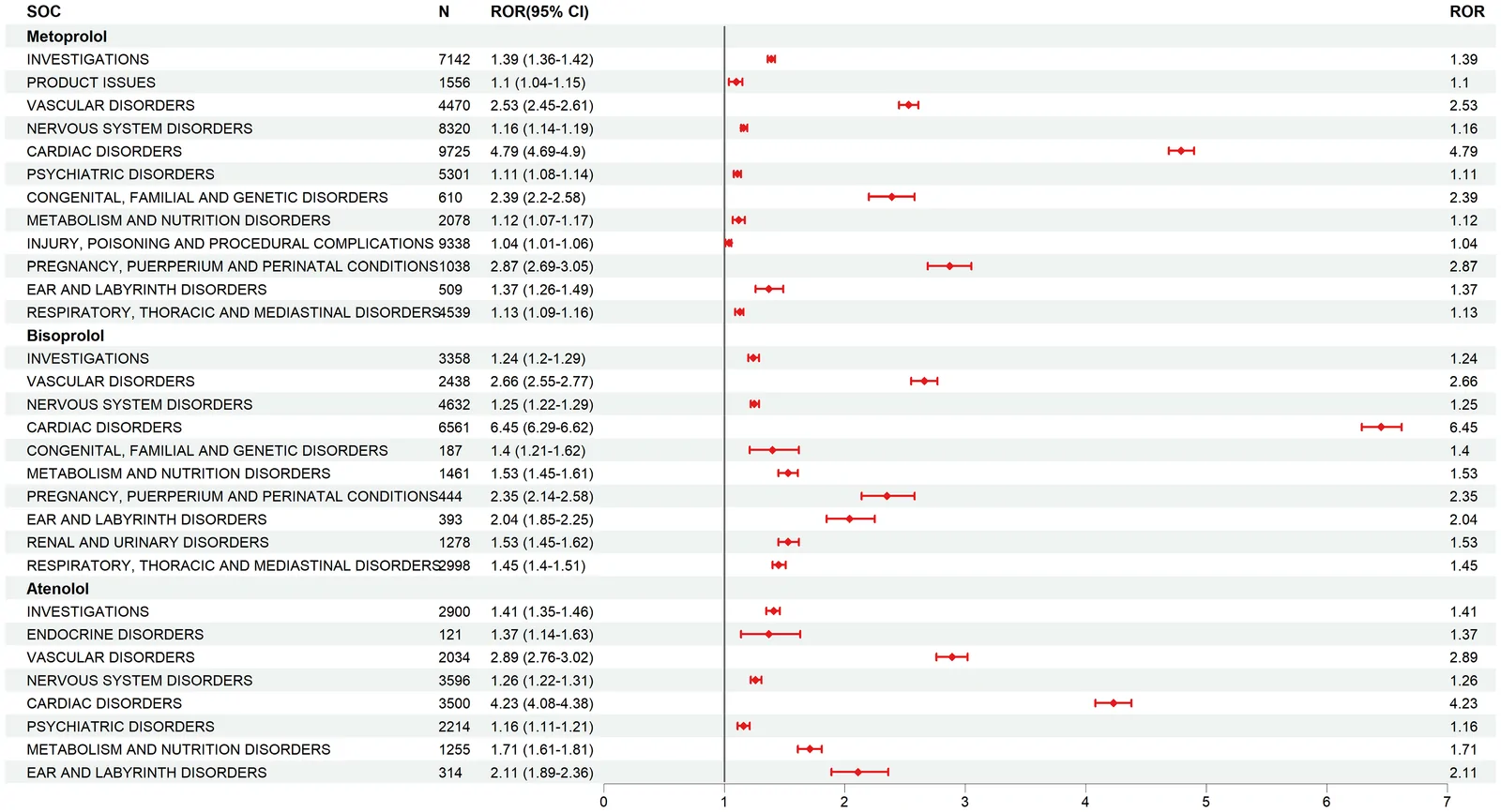
Forest plot of SOC-level disproportionality signals for metoprolol, bisoprolol, and atenolol. CI = confidence interval, SOC = system organ class.

For bisoprolol, the most prominent signal was likewise in cardiac disorders (ROR = 6.45), accompanied by increased risks in vascular disorders, metabolism and nutrition disorders, renal and urinary disorders, and ear and labyrinth disorders.

For atenolol, clear signals were similarly detected for cardiac disorders (ROR = 4.23) and vascular disorders (ROR = 2.89). In addition, atenolol exhibited relatively higher disproportionality in metabolism and nutrition disorders, ear and labyrinth disorders, and endocrine disorders.

Overall, all 3 β1-blockers shared a central pattern of enriched cardiovascular ADEs, particularly within cardiac disorders and vascular disorders. However, they differed in pregnancy-related, genetic, metabolic-endocrine, renal, and auditory SOC categories, reflecting heterogeneity in their broader safety profiles.

Figure 4 further illustrates these relationships using a Venn diagram, showing that metoprolol, bisoprolol, and atenolol share common SOC-level signals across cardiac disorders, vascular disorders, nervous system disorders, investigations, metabolism and nutrition disorders, and ear and labyrinth disorders, suggesting overlapping cardiovascular, neurologic, and metabolic safety characteristics.

**Figure 4.**
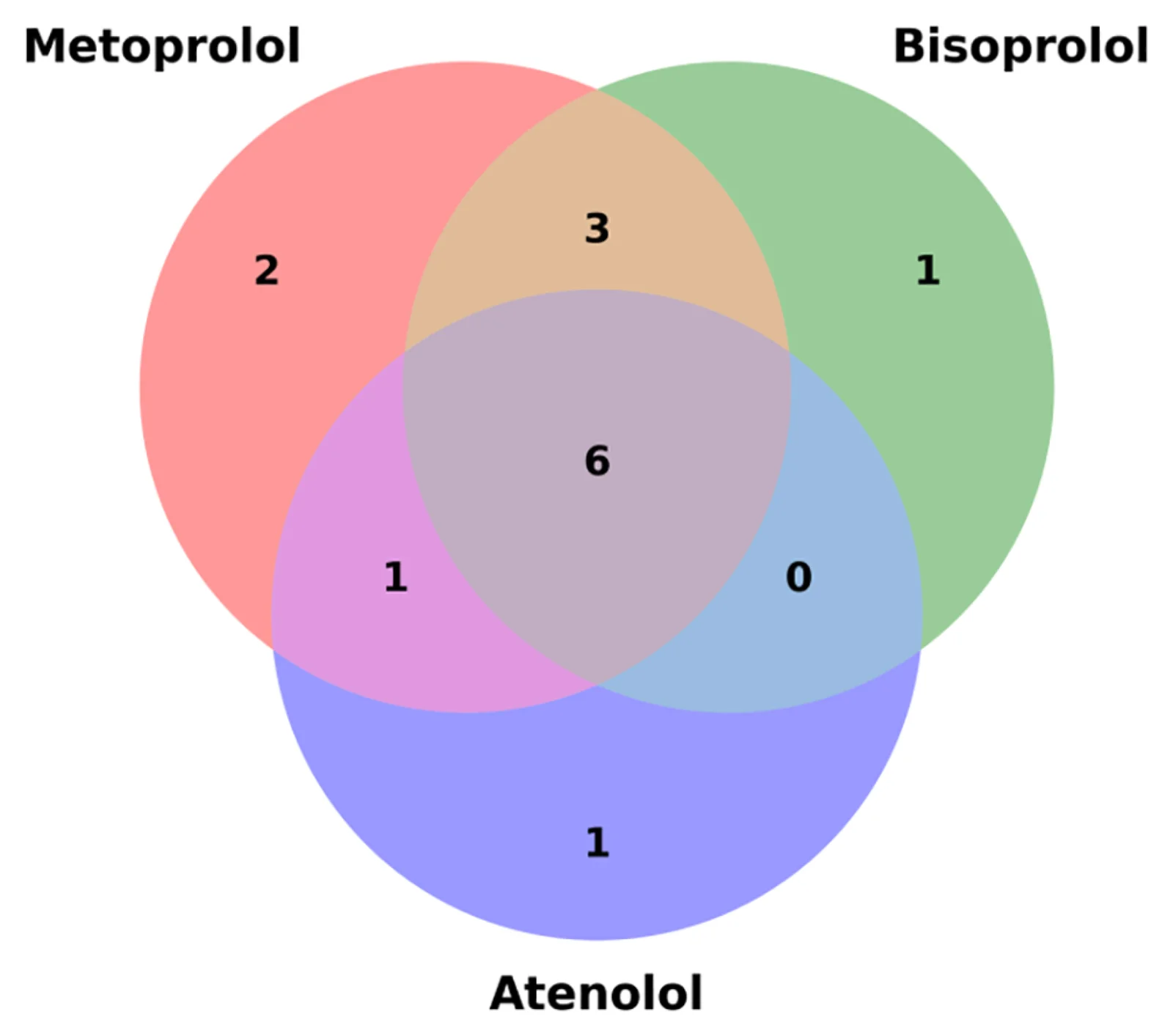
Venn diagram of overlapping SOC-level signals among the 3 selective β1-blockers. CI = confidence interval, SOC = system organ class.

Metoprolol and bisoprolol additionally shared signals in congenital, familial and genetic disorders, pregnancy, puerperium and perinatal conditions, and respiratory, thoracic and mediastinal disorders, whereas metoprolol and atenolol only overlapped in psychiatric disorders. Each drug also demonstrated several unique SOCs: Metoprolol – product issues, injury, poisoning and procedural complications; Bisoprolol – renal and urinary disorders; Atenolol – endocrine disorders. These distinct SOC-level features highlight subtle but clinically meaningful differences in the safety profiles of the 3 agents.

### 3.3. PT-level signal analysis

Figure 5 presents the forest plot of PT-level risk signals for selective β1-adrenergic receptor blockers.

**Figure 5.**
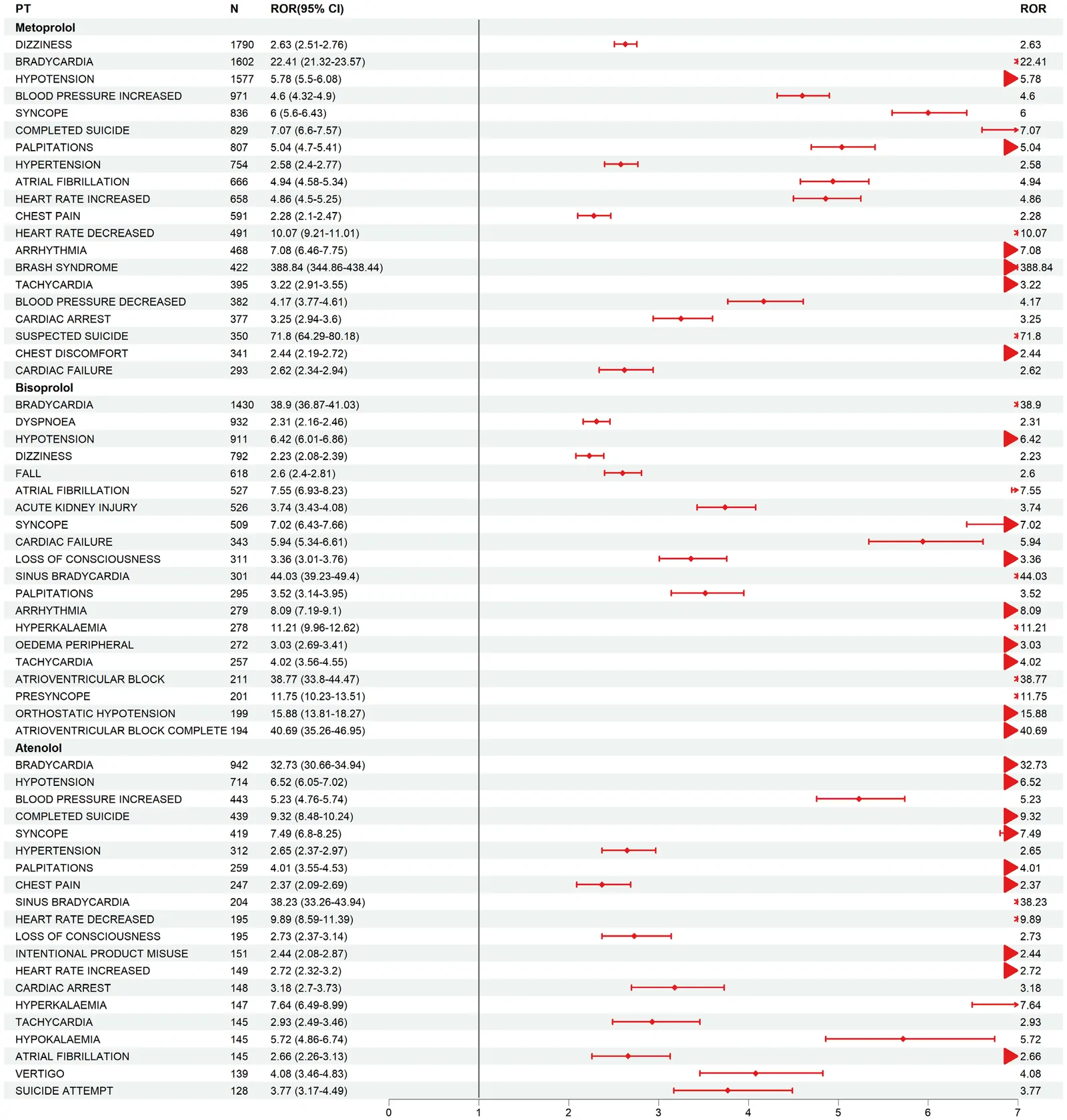
Forest plot of PT-level disproportionality signals for metoprolol, bisoprolol, and atenolol. PT = preferred term, ROR= reporting odds ratio.

For metoprolol, the most prominent signal was BRASH syndrome (ROR = 388.84, 95% confidence interval: 344.86–438.44), indicating highly disproportionate reporting but not a correspondingly large clinical effect or causal risk. Bradycardia-related and blood pressure-related PTs also generated signals, including bradycardia (ROR = 22.41), heart rate decreased (ROR = 10.07), and hypotension (ROR = 5.78). Additional signals were observed for syncope (ROR = 6.00), arrhythmia (ROR = 7.08), atrial fibrillation (ROR = 4.94), and tachycardia (ROR = 3.22). Suicide-related PTs, including completed suicide (ROR = 7.07) and suspected suicide (ROR = 71.80), were also disproportionately reported. These rare and clinically sensitive signals require independent verification because FAERS lacks the psychiatric history, indication, dose, and concomitant-treatment information needed for causal attribution.

For bisoprolol, the most notable reporting signals involved bradycardia and conduction abnormalities. Sinus bradycardia (ROR = 44.03), bradycardia (ROR = 38.90), atrioventricular block (ROR = 38.77), and atrioventricular block complete (ROR = 40.69) were disproportionately reported. Additional signals included orthostatic hypotension (ROR = 15.88), presyncope (ROR = 11.75), hyperkalaemia (ROR = 11.21), and acute kidney injury (ROR = 3.74). Other cardiac PTs included syncope (ROR = 7.02), arrhythmia (ROR = 8.09), atrial fibrillation (ROR = 7.55), and cardiac failure (ROR = 5.94). These measures characterize reporting disproportionality and should not be interpreted as incidence or comparative clinical risk.

For atenolol, the top 20 PTs were likewise dominated by bradycardia-related events. Sinus bradycardia (ROR = 38.23) and bradycardia (ROR = 32.73) showed disproportionate reporting. Blood pressure-related PTs included hypotension (ROR = 6.52) and blood pressure increased (ROR = 5.23). Atenolol was also associated with reporting signals for syncope (ROR = 7.49), heart rate decreased (ROR = 9.89), atrial fibrillation (ROR = 2.66), cardiac arrest (ROR = 3.18), hyperkalaemia (ROR = 7.64), and hypokalaemia (ROR = 5.72). Suicide-related PTs, including completed suicide (ROR = 9.32) and suicide attempt (ROR = 3.77), also generated signals. These findings warrant verification and cannot establish that atenolol caused the reported events.

We further conducted exploratory sex-stratified comparisons of PT reporting distributions. As shown in Table 2, nominal *P* values below .05 were observed for all 20 listed PTs associated with metoprolol. These comparisons were not adjusted for differences in age, indication, comorbidity, concomitant-treatment, or reporting behavior and should not be interpreted as causal sex effects.

**Table 2:** Sex-specific risk signals for metoprolol, bisoprolol, and atenolol.

Drug	Preferred term (PT)	Males with PT, n (a)	Males without PT, n (b)	Females with PT, n (c)	Females without PT, n (d)	*P* value
Metoprolol	Angina pectoris	98	31,970	94	43,298	.020
Metoprolol	Arthralgia	97	31,971	175	43,217	.026
Metoprolol	Atrial fibrillation	281	31,787	299	43,093	.004
Metoprolol	Brash syndrome	171	31,897	166	43,226	.003
Metoprolol	Cardiac arrest	177	31,891	169	43223	.001
Metoprolol	Cardiac failure	121	31,947	121	43,271	.021
Metoprolol	Confusional state	123	31,945	115	43,277	.005
Metoprolol	Death	140	31,928	122	43,270	<.001
Metoprolol	Dizziness	745	31,323	864	42,528	.002
Metoprolol	Dyspnoea	590	31,478	708	42,684	.032
Metoprolol	Fall	274	31,794	295	43,097	.007
Metoprolol	Heart rate decreased	205	31,863	210	43,182	.005
Metoprolol	Hypertension	233	31,835	432	42,960	<.001
Metoprolol	Inappropriate schedule of product administration	152	31,916	264	43,128	.016
Metoprolol	Insomnia	136	31,932	230	43,162	.044
Metoprolol	Myocardial infarction	178	31,890	166	43,226	<.001
Metoprolol	Nausea	262	31,806	475	42,917	<.001
Metoprolol	Product quality issue	130	31,938	233	43,159	.011
Metoprolol	Pruritus	118	31,950	212	43,180	.015
Metoprolol	Weight increased	110	31,958	201	43,191	.013
Bisoprolol	Adverse drug reaction	16	19,343	16	21,567	.896
Bisoprolol	Agitation	29	19,330	34	21,549	.942
Bisoprolol	Atrial fibrillation	231	19,128	255	21,328	.949
Bisoprolol	Atrioventricular block	86	19,273	100	21,483	.831
Bisoprolol	Bradycardia	597	18,762	664	20,919	.989
Bisoprolol	Cerebrovascular accident	23	19,336	27	21,556	.968
Bisoprolol	Diplopia	19	19,340	19	21,564	.863
Bisoprolol	Disorientation	23	19,336	23	21,560	.825
Bisoprolol	Epistaxis	23	19,336	28	21,555	.863
Bisoprolol	Hallucination	42	19,317	45	21,538	.938
Bisoprolol	Metabolic acidosis	19	19,340	23	21,560	.912
Bisoprolol	Myocardial infarction	44	19,315	47	21,536	.921
Bisoprolol	Oedema	24	19,335	29	21,554	.877
Bisoprolol	Pallor	24	19,335	28	21,555	.981
Bisoprolol	Product administration error	24	19,335	29	21,554	.877
Bisoprolol	Product dispensing error	19	19,340	20	21,563	.985
Bisoprolol	Rash pruritic	17	19,342	21	21,562	.879
Bisoprolol	Shock	22	19,337	22	21,561	.834
Bisoprolol	Somnolence	77	19,282	88	21,495	.935
Bisoprolol	Urinary incontinence	15	19,344	19	21,564	.843
Atenolol	Anxiety	38	12,140	115	20,297	.002
Atenolol	Atrioventricular block complete	46	12,132	38	20,374	.001
Atenolol	Blood glucose increased	42	12,136	45	20,367	.046
Atenolol	Cardiac arrest	62	12,116	69	20,343	.023
Atenolol	Chest pain	109	12,069	131	20,281	.012
Atenolol	Confusional state	54	12,124	60	20,352	.035
Atenolol	Cough	45	12,133	111	20,301	.034
Atenolol	Diarrhoea	54	12,124	144	20,268	.004
Atenolol	Drug hypersensitivity	40	12,138	121	20,291	.001
Atenolol	Drug interaction	141	12,037	184	20,228	.028
Atenolol	Feeling abnormal	46	12,132	147	20,265	<.001
Atenolol	Heart rate increased	40	12,138	102	20,310	.029
Atenolol	Insomnia	37	12,141	116	20,296	<.001
Atenolol	Intentional overdose	55	12,123	56	20,356	.010
Atenolol	Lethargy	43	12,135	29	20,383	<.001
Atenolol	Orthostatic hypotension	42	12,136	34	20,378	.002
Atenolol	Presyncope	51	12,127	44	20,368	.001
Atenolol	Product substitution issue	62	12,116	143	20,269	.041
Atenolol	Rash	39	12,139	113	20,299	.004
Atenolol	Toxicity to various agents	149	12,029	184	20,228	.006

*P* values refer to comparisons between males and females based on 2 × 2 contingency tables using 2-sided χ^2^ tests (or Fisher’s exact test where appropriate); *P* < .05 was considered statistically significant.

a, number of male reports with the specified PT.

b, number of male reports without the specified PT.

c, number of female reports with the specified PT.

d, number of female reports without the specified PT.

PT = preferred term.

Nominal *P* values below .05 were also observed for all 20 listed PTs associated with atenolol. These PTs included drug toxicity, drug interactions, chest pain, cardiac arrest, orthostatic hypotension, presyncope, anxiety, and insomnia. The findings are hypothesis-generating and require confirmation with prespecified multiplicity control and patient-level confounder adjustment.

In contrast, none of the 20 listed PTs associated with bisoprolol had a nominal *P* value below .05. Within this dataset, this indicates no detected difference in the reporting distribution between males and females for these selected PTs. It does not establish sex equivalence or a sex-neutral clinical safety profile.

### 3.4. Time-to-onset analysis of safety signals

To evaluate the temporal characteristics of adverse events associated with the 3 β1-adrenergic receptor blockers, Weibull TTO analyses were restricted to reports containing both a usable therapy-start date and a usable event-onset date. As shown in Table 3, a total of 22,651 metoprolol reports were included, with a median TTO of 78 days. For bisoprolol, 11,489 reports were analyzed, yielding a median onset time of 83 days. Atenolol had a longer median onset time of 174 days among 8928 analyzed reports. Reporter-provided dates may contain recall or documentation errors, so these estimates should be interpreted cautiously.

**Table 3:** Time to onset of metoprolol, bisoprolol, and atenolol adverse events and Weibull distribution analysis.

Drug name	time to onset (d)		Weibull distribution		
	Case reports	Median (d)	Scale parameter: α(95% CI)	Shape parameter: β(95% CI)	Curve type
Metoprolol	22,651	78	210.87 (191.58–230.16)	0.45 (0.44–0.47)	Early decline type
Bisoprolol	11,489	83	179.31 (164.09–194.01)	0.48 (0.47–0.51)	Early decline type
Atenolol	8928	174	373.71 (333.93–413.50)	0.49 (0.47–0.51)	Early decline type

CI = confidence interval, FAERS = US Food and Drug Administration Adverse Event Reporting System, TTO = time-to-onset.

In addition, the Weibull shape parameter β for all 3 agents was <1, and the corresponding 95% confidence intervals were also <1, indicating that the incidence of adverse events decreases over time. This pattern is characteristic of an early-failure type distribution.

From the distribution of onset times, adverse events associated with metoprolol and bisoprolol were primarily concentrated within the first 0 to 30 days after treatment initiation. In contrast, atenolol exhibited a distinctive pattern, with adverse events more commonly occurring after 360 days, as illustrated in Figure 6B–D.

**Figure 6.**
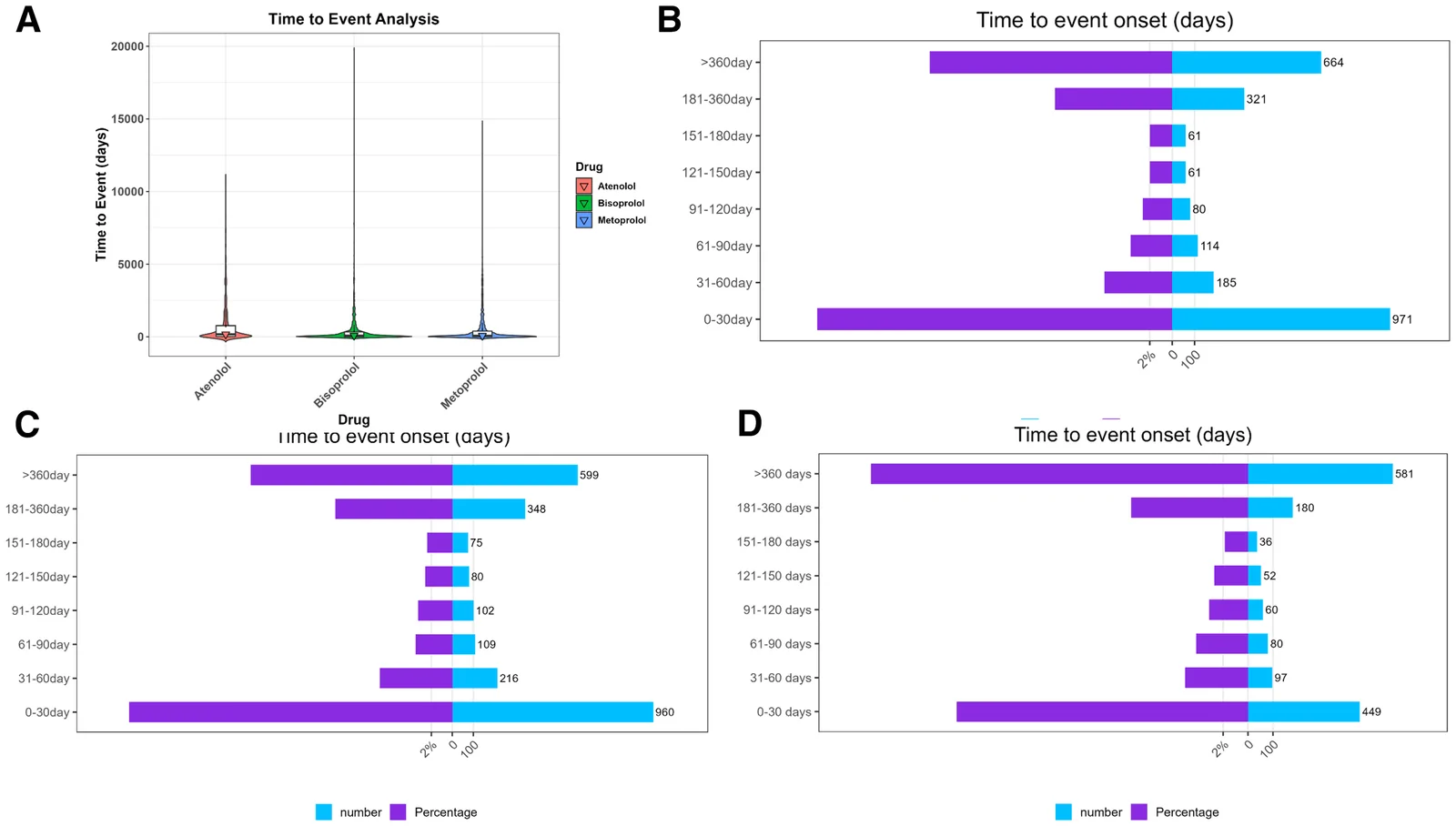
Time-to-onset distribution of adverse events associated with metoprolol, bisoprolol, and atenolol (A: violin plot; B–D: Weibull-based onset curves).

Furthermore, the violin plot in Figure 6A shows the presence of substantial outliers for all 3 drugs, suggesting considerable inter-individual variability in TTO distributions.

### 3.5. External validation using the Canadian CVARDD Database

To further ensure the accuracy and robustness of the findings, external validation was performed using adverse event data from the CVARDD.

Figure 7A presents the top 20 PT-level reporting patterns for metoprolol in FAERS and CVARDD. Cardiovascular events such as hypotension, bradycardia, and atrial fibrillation appeared prominently in both systems. Because the databases differ in size, population, and reporting practices, signal magnitudes were not interpreted as directly comparable. The overlap is therefore considered qualitative corroboration of the broad reporting spectrum rather than proof of reproducibility or causality.

**Figure 7.**
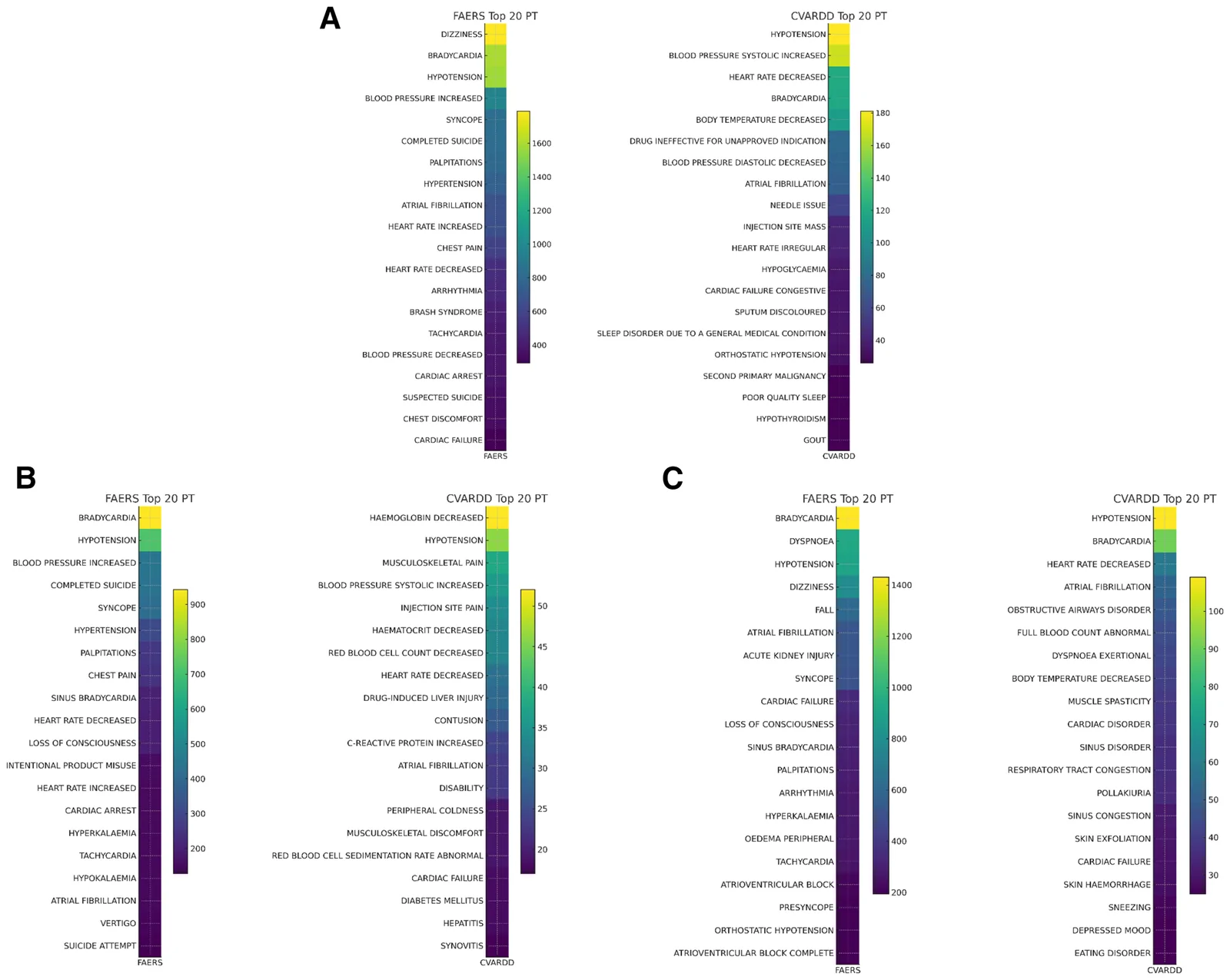
External validation using the CVARDD Database. (A) Heatmap of PT-level case counts for metoprolol in FAERS versus CVARDD; (B) Heatmap of PT-level case counts for bisoprolol in FAERS versus CVARDD; and (C) Heatmap of PT-level case counts for atenolol in FAERS versus CVARDD. CVARDD = Canadian Vigilance Adverse Reaction Online Database, FAERS = US Food and Drug Administration Adverse Event Reporting System, PT = preferred term.

Several non-cardiovascular PTs appeared in CVARDD, including injection-site reactions and second primary malignancy. These differences may reflect database-specific populations, coding, or reporting practices. Overall, CVARDD provided a complementary comparison of reporting patterns, while the limitations of cross-database spontaneous-report analyses preclude causal validation.

Figure 7B displays the top 20 PT-level reporting patterns for bisoprolol in both databases. Cardiovascular events, including bradycardia, hypotension, decreased heart rate, and atrial fibrillation, appeared prominently in both systems. This qualitative overlap supports further investigation of the observed reporting pattern but does not establish comparable incidence or clinical risk across databases.

Figure 7C shows the top 20 PT-level reporting patterns for atenolol. In both FAERS and CVARDD, cardiovascular events such as bradycardia, hypotension, syncope, atrial fibrillation, and decreased heart rate appeared in the prominent reporting spectrum.

Differences in database size and reporting practices may influence signal estimates and prevent direct quantitative comparison. CVARDD also contained hematologic and metabolic events, including decreased hemoglobin, reduced erythrocyte count, and elevated C-reactive protein. These observations may reflect system-specific or population-specific reporting and require independent confirmation.

## 4. Discussion

Using FAERS reports from 2004 through the second quarter of 2025, this study compared adverse event reporting patterns for metoprolol, bisoprolol, and atenolol, with a complementary comparison using CVARDD. Although these agents shared class-related reporting patterns, their observed signal spectra differed. These differences may reflect pharmacologic properties, but they may also arise from heterogeneity in indications, patient characteristics, co-medications, market exposure, and reporting behavior. The results should therefore be interpreted as hypothesis-generating pharmacovigilance signals rather than comparative estimates of clinical risk.

### 4.1. Adverse event characteristics and population differences

Our data show that more than 22,000 metoprolol, 11,000 bisoprolol, and 9000 atenolol adverse event reports were submitted globally, reflecting the extensive clinical use of these agents worldwide. Across all 3 drugs, the proportion of female patients exceeded that of males, and a high proportion of reports involved individuals older than 65 years, indicating that selective β1-blockers are predominantly used in older adults with multiple cardiovascular risk factors in real-world settings. This finding is consistent with previous evidence suggesting that elderly, high-risk, and multimorbid patients are key populations of concern regarding β-blocker safety.^[17]^

At the SOC level, all 3 agents generated signals in cardiac disorders and vascular disorders, with bradycardia, conduction disturbances, blood pressure fluctuations, and heart failure forming a shared reporting pattern. Signals were also observed in metabolism and nutrition disorders, nervous system disorders, and renal and urinary disorders. These findings identify areas for surveillance but do not establish that β1-blockers caused the reported events.^[9]^

At the PT level, all 3 drugs generated class-consistent signals for bradycardia, sinus bradycardia, hypotension, syncope, atrial fibrillation, and cardiac failure.^[6]^ The following drug-specific reporting patterns were observed:

• Metoprolol generated signals for BRASH syndrome and suicide-related events, including completed suicide and suspected suicide. These associations require cautious interpretation and independent verification.
• Bisoprolol generated signals for sinus bradycardia, atrioventricular block, hyperkalemia, and acute kidney injury.
• Atenolol generated signals involving blood pressure fluctuations, electrolyte disturbances, and toxicity-related or interaction-related PTs.

These patterns are biologically compatible with known pharmacologic differences, but the spontaneous-reporting design cannot establish the proposed mechanisms. Metoprolol is lipophilic and crosses the blood–brain barrier, yet the observed suicide-related reports may also reflect underlying psychiatric disease, indication, co-medication, and differential reporting.^[7]^ Bisoprolol undergoes partial renal elimination, and atenolol is predominantly renally excreted, which provides a rationale for monitoring renal function and electrolytes in susceptible patients.^[18-21]^ These mechanistic interpretations remain exploratory.

Clinically, the signals support attention to renal function, electrolytes, heart rate, blood pressure, symptoms, and co-medications when prescribing these agents. They do not establish that 1 drug is safer than another or justify treatment changes without consideration of approved indications, guideline recommendations, and the individual patient’s clinical context.

### 4.2. Sex differences and drug-specific risk profiles

Exploratory sex-stratified analyses identified differences in the reporting distributions of several PTs for metoprolol and atenolol, whereas no nominal differences were detected among the selected bisoprolol PTs. Because these comparisons were not adjusted for age, indication, comorbidity, co-medication, or multiple testing, they should not be interpreted as causal sex effects or as evidence that bisoprolol has a sex-neutral clinical safety profile. These observations should be interpreted alongside prior literature on sex differences in heart failure outcomes, cardiovascular risk, and responses to central nervous system drugs.^[22-24]^

Several biological and reporting mechanisms could contribute to these observations. Sex-related differences in body composition, baseline heart rate, autonomic activity, and drug-metabolizing enzyme expression may alter exposure at a given dose.^[25,26]^ Differences in symptom recognition and reporting may also affect spontaneous-report data.^[27]^ These explanations are exploratory and require confirmation in studies with denominator data, prespecified multiplicity control, and adjustment for clinical covariates.

The absence of nominal differences among the selected bisoprolol PTs should be interpreted cautiously. It may reflect the observed data, selection of the top PTs, sample composition, or reporting behavior rather than true equivalence between sexes.

These findings may help define monitoring questions for future study but should not determine drug choice or dose on their own. In clinical practice, β1-blocker selection and dosing should follow approved labeling and guidelines while considering age, renal function, blood pressure, heart rate, comorbidity, and concomitant therapy. Prospective or longitudinal studies are needed before sex-specific prescribing recommendations can be made from these signals. Related literature on thiazide-associated electrolyte disorders and falls, postmenopausal osteoporosis, and sex-linked myocardial injury mechanisms provides additional context for hypothesis generation.^[28-30]^

### 4.3. Time-to-onset characteristics and risk management

TTO analyses produced an early-failure Weibull pattern for all 3 agents. This pattern suggests that reported events were concentrated earlier after treatment initiation, but it does not estimate the time-varying incidence of adverse events. The longer median onset observed for atenolol may reflect treatment duration, missing or inaccurate dates, patient selection, or reporting behavior as well as pharmacologic factors.

The TTO findings support careful clinical review after treatment initiation and during dose titration. Monitoring should be individualized according to approved labeling, guideline recommendations, renal function, co-medications, and the patient’s baseline cardiovascular status. The present data do not support a fixed monitoring schedule or causal inference about the timing of drug effects. Background studies linking mental health with blood pressure and describing myocardial responses to beta-adrenergic stimulation further support individualized cardiovascular and symptom assessment, although they do not validate the present onset estimates.^[31,32]^

Furthermore, for patients receiving other heart rate – lowering or conduction-affecting medications (e.g., reversible cholinesterase inhibitors such as neostigmine, amiodarone, or non-dihydropyridine calcium channel blockers), rapid dose escalation should be avoided to reduce the risk of early severe bradycardia or cardiogenic shock.^[33]^

Substantial variability in reporter-provided onset times was observed. Late reports may occur during long-term therapy, but the spontaneous-reporting data cannot distinguish delayed drug effects from changes in disease, renal function, dose, adherence, or co-medication. Ongoing clinical assessment should therefore be guided by the patient’s condition rather than by the reported distributions alone.

### 4.4. Limitations

As with all pharmacovigilance studies based on spontaneous-reporting systems, this study has several limitations. FAERS lacks reliable exposure denominators and does not provide complete dose information, so true incidence rates, dose–response relationships, and comparative clinical risks cannot be estimated. Disproportionality measures identify reporting associations and cannot establish causality. Extreme ROR values, especially for rare PTs such as BRASH syndrome and suicide-related events, may be amplified by sparse counts, notoriety bias, stimulated reporting, and denominator differences. Suicide-related reports are additionally vulnerable to confounding by underlying psychiatric disease, indication, polypharmacy, and socioeconomic factors.

FAERS is also subject to underreporting, selective reporting, and residual duplication. Clinical variables such as body weight, renal function, psychiatric history, disease severity, indication, and concomitant medication are frequently missing. The 3 drug-report populations differed in age, indication, country, comorbidity, and likely prescribing context. These baseline differences can influence both the occurrence and reporting of events, and the present unadjusted analyses cannot separate drug-specific reporting patterns from population heterogeneity or prescription bias.

The sex-stratified comparisons were exploratory and were not adjusted for multiple testing or patient-level confounders. TTO analyses depended on reporter-provided therapy-start and event dates, which may contain recall or documentation errors, and information on dose changes, interruptions, and switching was incomplete. CVARDD provided a complementary cross-database comparison, but differences in database structure and reporting practices limit direct comparability. Future studies should use prespecified multiplicity control and adjusted analyses in electronic health records, claims data, or prospective cohorts to evaluate these hypotheses.

## 5. Conclusion

Using FAERS data from 2004 to the second quarter of 2025 and a complementary comparison with CVARDD, this study characterized adverse event reporting patterns for metoprolol, bisoprolol, and atenolol. The agents shared reporting signals involving bradycardia, conduction disorders, blood pressure fluctuations, and heart failure, while their broader signal spectra differed across metabolic, electrolyte, neuropsychiatric, and renal domains.

Metoprolol generated signals for BRASH syndrome and suicide-related events; bisoprolol for bradycardia, atrioventricular block, hyperkalemia, and acute kidney injury; and atenolol for blood pressure fluctuations, electrolyte abnormalities, and interaction-related events. These differences may reflect pharmacology, population heterogeneity, exposure, co-medication, and reporting practices. They should not be interpreted as causal effects or as direct rankings of clinical safety.

Exploratory sex-stratified comparisons and TTO analyses identified hypotheses for future research. The observed differences require confirmation with multiplicity control and adjustment for clinical covariates, and the onset patterns remain sensitive to missing or inaccurate dates.

Selective β1-adrenergic receptor blockers remain important cardiovascular therapies. The present signals may help focus monitoring and future confirmatory studies, but drug selection and dosing should continue to follow approved labeling, clinical guidelines, and individual patient characteristics. Longitudinal studies with reliable exposure denominators and detailed covariate data are needed to estimate comparative risk and evaluate causality.

## Acknowledgments

This study used publicly available data from the FAERS and Canadian Vigilance databases. We thank the reporters and organizations that contributed to these pharmacovigilance systems.

## Author contributions

**Conceptualization:** Peili Wang.

**Data curation:** Weijie Ren, Haosen Zhao, Zhenzhong Wang, Zezhou Zhang, Peili Wang.

**Funding acquisition:** Jixin Li, Peili Wang.

**Methodology:** Weijie Ren, Zhenzhong Wang, Jixin Li, Zelong Niu, Ruiling Zhou, Zhijie Cui, Zezhou Zhang, Peili Wang.

**Resources:** Zezhou Zhang.

**Software:** Weijie Ren, Haosen Zhao, Zhenzhong Wang, Jixin Li, Zelong Niu, Ruiling Zhou, Zhijie Cui, Zezhou Zhang.

**Validation:** Zhenzhong Wang.

**Visualization:** Weijie Ren, Haosen Zhao.

**Writing – original draft:** Weijie Ren, Haosen Zhao, Zelong Niu, Zezhou Zhang, Peili Wang.

**Writing – review & editing:** Weijie Ren, Haosen Zhao, Zhenzhong Wang, Jixin Li, Ruiling Zhou, Zhijie Cui, Peili Wang.


