# Supplementary material for: Comparative real-world safety profiles of selective β1-adrenergic receptor blockers: A multi-database pharmacovigilance disproportionality study
Source: Medicine (Baltimore). 2026 Sep 25;105(39):e50763. doi: 10.1097/MD.0000000000050763 (PMC13619275; doi:10.1097/MD.0000000000050763)
Supplement: Supplementary file 1 [file medi-105-e50763-s001.docx]

**Supplementary material 1** Two-by-two contingency table for disproportionality analyses.

| Drugs | Target AEs | Other AEs |
| --- | --- | --- |
| Target drugs | a | b |
| Other drugs | c | d |
| Total | a+c | b+d |
| Algorithms | Equation | Criteria |
| ROR | ROR=ad/b/c | lower limit of 95% CI>1, N≥3 |
|  | 95%CI=e^ln(ROR)±1.96(1/a+1/b+1/c+1/d)^0.5^ |  |
| PRR | PRR=a(c+d)/c/(a+b) | PRR≥2, χ^2^≥4, N≥3 |
|  | χ^2^=[(ad-bc)^2](a+b+c+d)/[(a+b)(c+d)(a+c)(b+d)] |  |
| BCPNN | IC=log_2_a(a+b+c+d)(a+c)(a+b) | IC025>0 |
|  | 95%CI= E(IC) ± 2V(IC)^0.5 |  |
| EBGM | EBGM=a(a+b+c+d)/(a+c)/(a+b) | EBGM05>2 |
|  | 95%CI=eln(EBGM)±1.96(1/a+1/b+1/c+1/d)^0.5 |  |

Abbreviation: AEs, adverse events; a, number of reports containing both the target drug and target adverse drug reaction; b, number of reports containing other adverse drug reaction of the target drug; c, number of reports containing the target adverse drug reaction of other drugs; d, number of reports containing other drugs and other adverse drug reactions. 95%CI, 95% confidence interval; N, the number of reports; χ2, chi-squared; IC, information component; IC025, the lower limit of 95% CI of the IC; E(IC), the IC expectations; V(IC), the variance of IC; EBGM, empirical Bayesian geometric mean; EBGM05, the lower limit of 95% CI of EBGM.
